# Integrated genomic analysis identifies the mitotic checkpoint kinase WEE1 as a novel therapeutic target in medulloblastoma

**DOI:** 10.1186/1476-4598-13-72

**Published:** 2014-03-24

**Authors:** Peter S Harris, Sujatha Venkataraman, Irina Alimova, Diane K Birks, Ilango Balakrishnan, Brian Cristiano, Andrew M Donson, Adrian M Dubuc, Michael D Taylor, Nicholas K Foreman, Philip Reigan, Rajeev Vibhakar

**Affiliations:** 1 Department of Pediatrics and Section of Pediatric Hematology/Oncology/BMT, Children's Hospital Colorado and University of Colorado Denver, Anschutz Medical Campus, 12800 E 19th Ave, Mail Stop 8302, Aurora, CO, 80045, USA; 2 Department of Neurosurgery, Children's Hospital Colorado and University of Colorado Denver, Anschutz Medical Campus, 12800 E 19th Ave, Mail Stop 8302, Aurora, CO, 80045, USA; 3 Department of Pharmaceutical Sciences, Skaggs School of Pharmacy and Pharmaceutical Sciences, University of Colorado Denver, Anschutz Medical Campus, 12850 E Montview Blvd, Mail Stop C238, Aurora, CO, 80045, USA; 4 Division of Neurosurgery, Arthur & Sonia Labatt Brain Tumour Research Centre, The Hospital for Sick Children, 555 University Ave, Rm 1503, Toronto, ON, M5G 1X8, Canada

**Keywords:** Medulloblastoma, *WEE1*, Mitosis, MK-1775, Integrated genomics

## Abstract

**Background:**

Medulloblastoma is the most common type of malignant brain tumor that afflicts children. Although recent advances in chemotherapy and radiation have improved outcomes, high-risk patients do poorly with significant morbidity.

**Methods:**

To identify new molecular targets, we performed an integrated genomic analysis using structural and functional methods. Gene expression profiling in 16 medulloblastoma patient samples and subsequent gene set enrichment analysis indicated that cell cycle-related kinases were associated with disease development. In addition a kinome-wide small interfering RNA (siRNA) screen was performed to identify kinases that, when inhibited, could prevent cell proliferation. The two genome-scale analyses were combined to identify key vulnerabilities in medulloblastoma. The inhibition of one of the identified targets was further investigated using RNAi and a small molecule inhibitor.

**Results:**

Combining the two analyses revealed that mitosis-related kinases were critical determinants of medulloblastoma cell proliferation. RNA interference (RNAi)-mediated knockdown of *WEE1* kinase and other mitotic kinases was sufficient to reduce medulloblastoma cell proliferation. These data prompted us to examine the effects of inhibiting *WEE1* by RNAi and by a small molecule inhibitor of WEE1, MK-1775, in medulloblastoma cell lines. MK-1775 inhibited the growth of medulloblastoma cell lines, induced apoptosis and increased DNA damage at nanomolar concentrations. Further, MK-1775 was synergistic with cisplatin in reducing medulloblastoma cell proliferation and resulted in an associated increase in cell death. *In vivo* MK-1775 suppressed medulloblastoma tumor growth as a single agent.

**Conclusions:**

Taken together, these findings highlight mitotic kinases and, in particular, *WEE1* as a rational therapeutic target for medulloblastoma.

## Introduction

Medulloblastoma is the most common malignant brain tumor in children. Over 800 cases per year occur worldwide with the vast majority occurring in children less than 12 years of age [1]. The mainstays of medulloblastoma therapy continue to be surgery, radiation and cytotoxic chemotherapy [2]. While therapy for standard risk patients has resulted in improved outcomes, high-risk patients do poorly. In particular those showing relapse and *MYC* amplification have a 5-year survival rate of less than 40% [3]. In addition, there remains significant therapy-related morbidity, particularly in the very young patients [4-6]. Novel therapeutic approaches based on tumor biology are clearly needed to improve outcomes for these children.

Recent genomic analysis has been successfully used to identify medulloblastoma subtypes [7-10]. International consensus has resulted in four molecular subgroups being defined [11]. These are the Wnt and Shh signaling subgroups as well as Group 3 and 4. Group 3 tumors largely represent the *MYC* amplified tumors whereas there is not a clear molecular definition of the Group 4 tumors [11]. However, finding therapeutic targets from these categories is still challenging [12]. Patients with the Wnt signaling signature are in a very good risk category and efforts are underway to de-escalate therapy for this cohort of patients [13]. For patients with the Shh signature, there are targeted inhibitors currently in early phase trials [13]. Unfortunately molecular targeting for Group 3 and 4 tumors is less clear. This is particularly problematic since Group 3 and 4 tumors constitute 60% of all medulloblastoma tumors [11].

The advent of RNA interference (RNAi) technologies for targeting large sets of genes in mammalian cells allows us to systematically interrogate gene functions in a high throughput manner [14,15]. This functional genomic approach has successfully resulted in the discovery of genes that were components of Ras oncogene driven tumors [16,17], of genes that sensitize cells to chemotherapeutic agents [18], and of genes essential to the proliferation of such diverse cancer cells as neuroblastoma and renal cell carcinoma [19,20].

Here we use an integrated descriptive and functional genomic analysis to identify molecular targets for medulloblastoma therapy. We performed pathway and gene set enrichment analysis on expression profiling data from 16 medulloblastoma samples to identify potential targetable pathways. In conjunction we performed a kinome-wide siRNA screen of medulloblastoma cells. Combined these results identified a set of mitotic-related kinases as potential therapeutic targets for medulloblastoma. We show that genetic and chemical inhibition of one of these kinases, *WEE1*, potently suppresses cell growth, induces apoptosis and decreases tumor volume *in vivo* in medulloblastoma. Further a small molecule inhibitor, MK-1775, acts in synergy with cisplatin to induce medulloblastoma cell death *in vitro*.

## Materials and methods

### Cell lines and primary patient samples

Dr. Darell D. Bigner (Duke University Medical Center, NC) kindly provided the D425 and D458 medulloblastoma suspension cell lines. The ONS-76 medulloblastoma cell line was graciously given by Dr. James T. Rutka (University of Toronto, Canada) and the UW228 cell line by Dr. John Silber (University of Washington, Seattle). The Daoy and D283 medulloblastoma cell lines were purchased from American Type Cell Culture (Rockville, MD). All cell lines were cultured in DMEM (Gibco, Carlsbad, CA) supplemented with 10% fetal bovine serum (Atlanta Biologicals, Lawrenceville, GA).

**Figure 1:**
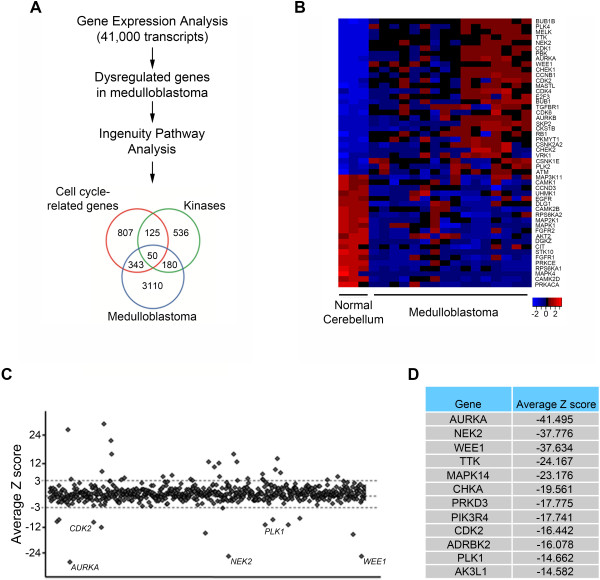
**Analysis of cell cycle-related kinases in medulloblastoma. (A)** Schematic of the integrated genomic analysis undertaken to identify novel targets in medulloblastoma. This approach identified 50 potential cell cycle-related kinases in medulloblastoma. **(B)** Heat map showing the gene expression of the 50 identified dysregulated cell cycle-related kinases in 16 medulloblastoma patient samples compared to three normal cerebellum samples. **(C)** Dot plot showing the average Z score for the 710 human kinase genes targeted in the siRNA screen. Each dot represents the average Z score of the 3 separate siRNAs targeting that single kinase. **(D)** The table lists the top 12 kinase genes with the lowest Z score from the siRNA screen performed in Daoy medulloblastoma cells.

**Figure 2:**
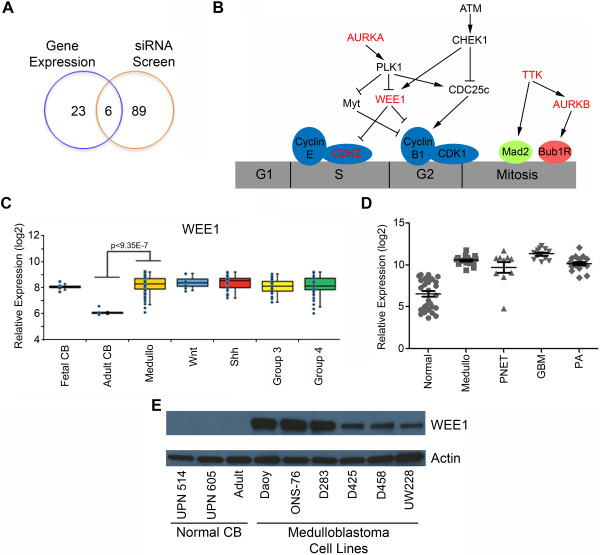
**Mitotic kinases as therapeutic targets in medulloblastoma. (A)** The Venn diagram shows the overlap of 29 kinases identified by gene expression analysis to have high expression in medulloblastoma with 95 kinases found to be important for medulloblastoma proliferation in the siRNA kinase screen. *WEE1* was one of the 6 kinases in common between the two approaches. **(B)** Model of G2-M kinases that mediate Daoy cell proliferation. Kinases in red are the hits from the combination of gene expression analysis and the kinome-wide RNAi screen. **(C)***WEE1* mRNA expression in 90 medulloblastoma patient samples shows a significant increase when compared to normal adult cerebellum (Adult CB) by microarray analysis. There is no significant difference in *WEE1* expression between the 4 subgroups of medulloblastoma (Wnt, Shh, Group 3 and Group 4). **(D)** Microarray analysis shows an increase in *WEE1* mRNA expression in a cohort of pediatric medulloblastoma (Medullo), primitive neuroectodermal tumors (PNET), glioblastoma multiforme (GBM) and pediatric pilocytic astrocytoma (PA) when compared to normal brain specimens. **(E)** WEE1 protein levels are increased in six common medulloblastoma cell lines. UPN 514 and UPN 605 are from normal pediatric cerebellum lysates (Normal CB).

The discovery cohort of 16 medulloblastoma patient samples used for gene expression profiling in Figure 1B and additional patient samples representing primitive neuroectodermal tumor, glioblastoma multiforme and pilocytic astrocytoma were obtained from Children’s Hospital Colorado and was conducted in accordance with local and federal human research protection guidelines and Institutional Review Board (IRB) regulations. Informed consent was obtained for all specimens collected. Normal cerebellum collected from autopsy was purchased from Ambion (Austin, TX), Stratagene (Santa Clara, CA) and Clontech Laboratories, Inc. (Mountain View, CA). The other normal cerebellar samples (UPN 514 and UPN 605) were obtained from nonmalignant brain biopsies at the Children’s Hospital Colorado under IRB guidelines. UPN 514 and UPN 605 are from 4 year old and 5 year old patients, respectively. The second large cohort of 90 tumor specimens were obtained in accordance with the Research Ethics Board at the Hospital for Sick Children (Toronto, Canada) and the N. N. Burdenko Neurosurgical Institute (Moscow, Russia) and data analysis was performed as described [7]. The second cohort was used to generate *WEE1* gene expression array data for the normal cerebellum and the four distinct medulloblastoma molecular subgroups given in Figure 2C.

### Transfections with RNAi

The siPORT NeoFX Transfection Agent purchased from Ambion was used to transfect the siRNAs targeting *WEE1* mRNA (s21) and a non-targeting siRNA into medulloblastoma cell lines at a final concentration of 5 nM. The manufacturer’s suggested protocol for a reverse transfection was used with the siRNA.

### Small molecule inhibitor of WEE1

The small molecule WEE1 inhibitor, MK-1775, was purchased from Axon Medchem (Groninberg, Netherlands) or synthesized by us. Dimethyl sulfoxide (DMSO) was used to reconstitute the drug and subsequent aliquots were stored in a desiccator at -20°C. An equivalent amount of DMSO for the highest concentration of drug was used for each experiment as a vehicle control.

### Cell proliferation assays

Cell growth was measured using the xCELLigence system and E-Plate 96-well gold-coated plates (Roche, Indianapolis, IN). This system gives the real-time measurement of cell proliferation [21]. Cells were transfected with a siRNA against *WEE1* or with a non-targeting siRNA control for 48 hours. Cells were then trypsinized and 2000 cells were plated in to a well of an E-plate and cell growth was measured.

Cell proliferation was determined by MTS [3-(4, 5-dimethylthiazol-2-yl)-5-(3- carboxymethoxyphenyl)-2-(4-sulfophenyl)-2H-tetrazolium] assay using CellTiter 96 AQueous One Solution (Promega, Madison, WI). For RNAi experiments 20 μL of MTS reagent was added to wells containing 100 μL volumes seventy-two hours post-transfection. For chemical inhibitor experiments cells were plated for 24 hours before adding MK-1775. Then 72 hours after the addition of the drug, 30 μL of MTS reagent was added to the wells to make a final volume of 180 μL. The optical densities of the triplicate wells were read using a BioTek Synergy 2 plate reader (Winooski, VT) every hour for 4 hours after the addition of the MTS reagent. Background absorbance was subtracted from all wells before analysis. The half maximal inhibitory concentration (IC50) values for the drug were calculated from the corrected absorbance values using GraphPad Prism.

### Colony formation assay

Cells were initially transfected with siRNA and then after 48 hours were counted and replated at 500 cells per well of a 6-well plate in triplicate. Colony formation assays utilizing MK-1775 were plated at the same density as above for 24 hours before drug addition. Wells were then treated with drug for 24 hours and subsequently allowed to grow in normal culture medium. Cells were allowed to grow for seven days, then the medium was aspirated, the wells were washed with phosphate buffered saline (PBS) and the colonies were stained with 0.5% crystal violet/25% methanol solution. A dissecting microscope was used to count the number of colonies per well with a threshold of 50 cells necessary to constitute a colony.

### Cell apoptosis assay

Cells were treated for 24 hours with drug and then allowed to grow in normal culture medium for an additional 24 hours. The cell concentration was determined following staining with Guava ViaCount reagent (Millipore, Billerica, MA). Equal numbers of cells were then stained using Guava Nexin reagent (Millipore) to detect apoptotic cells. Samples were run on a Guava EasyCyte Plus flow cytometer (Millipore).

### Immunofluorescence

Three thousand cells grown in poly-D-lysine coated chamber slides were treated with either an IC25 of cisplatin, an IC30 of the WEE1 inhibitor MK-1775, cisplatin plus MK-1775 or DMSO for 6 hours or 24 hours. After treatment cells were washed and fixed with 4% paraformaldehyde for 15 minutes at room temperature. Cells were then permeabilized with 0.2% Triton X-100 in PBS for 15 minutes followed by incubation in 5% milk diluted in 0.05% Triton X-100 for 30 minutes at room temperature on a shaker. After blocking, cells were incubated with the primary antibodies. The following antibodies were used at a dilution of 1:200: anti-γH2AX (Ser139), anti-phospho-H3 (Ser10) or anti-caspase 3 for 1 hour at room temperature. After washing with 0.05% Triton X-100 (3 times for 5 minutes each) cells were incubated with Alexa Fluor 488 conjugated secondary antibody (1:500) for 1 hour at room temperature in the dark, washed with PBS (3 times for 5 minutes each) and mounted using ProLong Gold antifade reagent containing DAPI (Invitrogen). Images were acquired using an inverted epifluorescence microscope at a magnification of 20x. At least three random fields were chosen to count cells containing greater than 10 foci.

### RNAi kinase screen

A siRNA library containing 3 siRNAs per kinase for 710 human kinase genes was purchased from Ambion (catalog #4397918). The siRNAs in the 96-well plates were resuspended in nuclease-free sterile water to a concentration of 1 μM. Aliquots of the siRNAs were further diluted into daughter plates at a concentration of 250 nM. Daoy cells from the same passage were used to perform the screen. Briefly, Daoy cells were reverse transfected using siPORT NeoFX Transfection Agent with a final siRNA concentration of 5 nM. A separate triplicate set of wells containing cells transfected with a non-silencing siRNA were included on each plate. A MTS assay was performed 72 hours after transfection to determine whether the particular gene targeted by the siRNA had any effect on cell proliferation.

### Western blotting

RIPA buffer (Thermo Scientific, Rockford, IL) containing protease inhibitors was used to obtain protein lysates. Western blotting was performed per standard methods. Primary antibodies purchased from Cell Signaling Technology that were used are WEE1 (#4936), γH2AX (#2577), phospho-CDK1 (Tyr15) (#4539) and CDK1 (#9112). The primary antibodies against actin (MAB1501) purchased from Millipore or β-tubulin (MAB1637) from Chemicon were used as loading controls. Secondary antibodies conjugated to horseradish-peroxidase were used in conjunction with a chemiluminescent reagent to visualize protein bands.

### Gene expression analysis

Patient tumor samples were evaluated for gene expression using Affymetrix U133 Plus 2.0 Gene Chip microarrays as previously described [22]. Briefly, samples were collected at the time of surgery and snap-frozen in liquid nitrogen. RNA was extracted from each sample using an RNeasy kit (Qiagen, Valencia, CA, USA), reverse-transcribed and the resulting cDNA was converted to cRNA, and hybridized to HG-U133 Plus 2.0 GeneChips (Affymetrix) according to the manufacturer’s instructions. Data analysis was performed in R (http://www.r-project.org/) using packages publicly available through Bioconductor (http://www.bioconductor.org). Hierarchical clustering was performed using the normalized gene expression data. Functional annotation analysis of differentially expressed genes was performed with the NIH Database for Annotation, Visualization, and Integrated Discovery (DAVID) Web tool (http://david.abcc.ncifcrf.gov/) using Biological Process Gene Ontology (GO) terms and Kyoto Encyclopedia of Genes and Genomes (KEGG) pathways. Gene set enrichment analysis was used to examine enrichment of genes in predefined reference sets that are based on biological knowledge [23]. Unlike other approaches that examine only genes meeting a predetermined cutoff, this tool computes an aggregate score for all genes in the reference set, based on their relative ranking in the data. Functional network analysis was performed using Ingenuity Pathways Analysis (IPA; Ingenuity Systems; http://www.ingenuity.com) which enables the visualization and exploration of molecular interaction networks on the basis of gene expression data. Networks were then algorithmically generated on the basis of their connectivity and were ranked by IPA on the basis of the number of genes represented in the network from the submitted gene list.

### *In vivo* xenograft analysis

This study was conducted at Proxy Bioresearch, Inc. at its AAALAC accredited facility Proxy Bio, S.A. de C.V. The study was conducted following industry standards including compliance to United States Department of Agriculture and NIH animal care and use guidelines. Athymic nude mice were selected for this study because they have been used for similar tumor grafting studies and have been well-characterized. Daoy cell pellets were prepared using standard harvest procedures, resuspended in PBS and 7 × 10^7^ cells were injected with 50% Matrigel by volume (BD Matrigel) using a 1 mL syringe and 18 gauge 1/2 inch needle into the flank of each mouse in an approximately 200 μL volume. Once tumors were palpable and measurable (mean 52 days post inoculation) treatment with MK-1775 or the control vehicle of DMSO was begun.

Control or MK-1775 (30 mg/kg) was dosed for 3 consecutive days every 7 days for 3 weeks by oral gavage and then followed for an additional 42 days with no therapy given. Each animal was tracked individually for tumor growth by external caliper measurements of subcutaneous protruding tumor and an approximate tumor volume was calculated using the ellipsoid volume formula: π/6 × L × W × H. Animals were also weighed 3 times a week for the duration of the treatment. Upon termination of the study the tumors were excised and snap frozen in liquid nitrogen.

### Cell cycle analysis

Cells were treated with an IC30 of MK-1775, an IC25 of cisplatin, or both for 24 hours. Cells were then harvested, washed with PBS and fixed in 70% ethanol. Twenty-four hours after fixing cells were washed with PBS and then resuspended in Guava Cell Cycle reagent and analyzed on a Guava EasyCyte Plus flow cytometer.

### Statistics

The p value for the gene expression of the second large cohort of 90 patient samples was calculated using the Mann-Whitney U test. The remaining p values were calculated using the Student’s t test. The error bars represent the standard error of the mean.

## Results

### Cell cycle-related kinases are differentially expressed in medulloblastoma

As a first step to identify novel molecular targets we performed gene expression profiling on 16 medulloblastoma samples and on corresponding normal cerebellar tissue. The tumor samples were obtained at our institution at the time of surgery and represent all major molecular subgroups as previously described by us [24]. Gene expression was measured by Affymetrix microarrays [24]. We selected for dysregulated genes with a two-fold or greater difference between medulloblastoma and normal cerebellum (FDR 0.1). A total of 3683 transcripts were differentially expressed in medulloblastoma. To identify specific signaling networks we performed pathway analysis using IPA software (Ingenuity) and gene set enrichment analysis. Cell cycle-related genes were the most abundant in the molecular category and kinases were the most abundant in the functional category. By comparing the molecular and functional categories with the total dysregulated genes in medulloblastoma we found 50 specific genes that were common to all three categories (Figure 1A). The expression of these cell cycle-related kinases is shown in the heat map in Figure 1B and in Additional file 1: Table S1. Twenty-nine of the fifty dysregulated cell cycle-related kinases are significantly over expressed compared to normal cerebellum.

### Role of protein and lipid kinases in medulloblastoma cell proliferation

Based on our initial data that a subset of kinases are over expressed in medulloblastoma we carried out a kinome-wide siRNA screen to identify kinases that are essential for medulloblastoma cell proliferation. The well characterized Daoy cell line was chosen as we have significant previous experience in manipulating it [24,25]. Daoy cells were transfected with 2130 unique siRNAs targeting each of 710 kinase genes or a non-silencing control in a 96 well format. Cell proliferation was evaluated by the MTS assay (CellTiter AQueous) after 72 hours of transfection. Absorbance values were normalized to reference controls on each plate to allow plate to plate comparison and the average Z score was calculated (Figure 1C). A Z score of < -2 was considered effective on decreasing medulloblastoma proliferation. A total of 95 genes were identified that when inhibited decreased Daoy cell growth (Figure 1C and Additional file 2: Table S2). Fifty genes met our criteria when we use a more stringent cutoff of Z < -3 with the 12 genes pertaining to the lowest Z score shown in Figure 1D. Of these we have previously reported on polo-like kinase 1 and aurora kinase A [25,26]. We subsequently validated five of these genes in an independent siRNA screen for their effect on medulloblastoma cell proliferation (Additional file 3: Figure S1). Of note as expected not all siRNA resulted in a biological phenotype due to the variability of siRNA knockdown in cells.

### Mitotic kinases are key determinants of medulloblastoma cell growth

To integrate our gene expression data and our siRNA screen we compared the two sets of data. The 29 genes over expressed from the gene expression data was compared to the 95 kinases identified to inhibit cell proliferation by the siRNA screen. Only 6 genes are over expressed in medulloblastoma and also suppress medulloblastoma cell growth. These are *aurora kinase A*, *WEE1*, *TTK*, *aurora kinase B*, *CDK2* and *PLK1* (Figure 2A). Interestingly all 6 genes are involved in the G2-M transition during the cell cycle (Figure 2B). Our previous report on the importance of aurora kinase A in medulloblastoma cell proliferation further validates this new methodology of identifying genes by integrated genomic analysis [26,25]. We chose to further examine the role of WEE1 kinase because it has recently been reported to be involved in several tumors including glioblastoma [27]. WEE1 regulates the G2-M cell cycle checkpoint by preventing DNA damaged cells from entering mitosis. We first examined the expression of *WEE1* in an independent cohort of 90 medulloblastoma samples. Comparison of normal cerebellum with medulloblastoma showed significant over expression of *WEE1* (p < 9.35E-7) in this disease. However there was no significant difference in *WEE1* expression between the recently identified subgroups as shown in Figure 2C. Next *WEE1* expression was examined in a panel of pediatric brain tumors of differing pathological grade. *WEE1* was over expressed in all three high-grade tumors examined including medulloblastoma (medullo), primitive neuroectodermal tumor (PNET) and glioblastoma multiforme (GBM). Interestingly *WEE1* was also expressed in the low-grade pilocytic astrocytoma (PA) in which there is very little proliferation (Figure 2D). These data suggested that increased *WEE1* expression may be a key to tumorigenesis and not simply a marker of proliferation. We next evaluated WEE1 protein in two normal pediatric cerebellum samples, one adult normal cerebellum sample and in a panel of well characterized medulloblastoma cell lines. WEE1 protein is not present in pediatric (UPN 514, 605) or adult cerebellum but was present in varying amounts in the 6 medulloblastoma cell lines evaluated (Figure 2E).

### Inhibition of WEE1 suppresses medulloblastoma cell proliferation and colony forming potential in vitro

**Figure 3:**
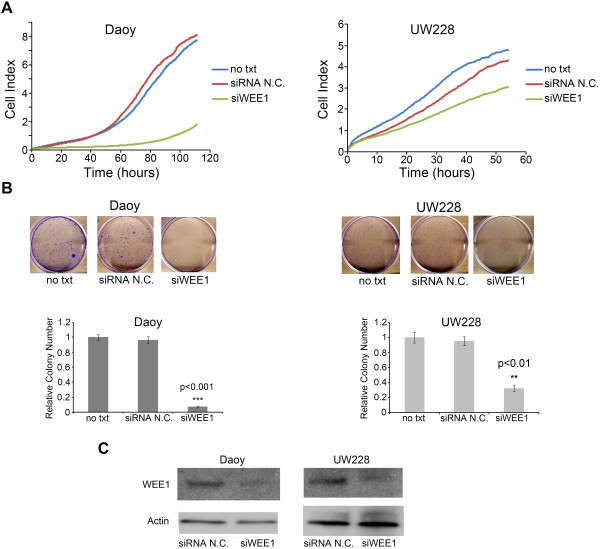
**siRNA-mediated inhibition of *WEE1 *decreases medulloblastoma cell proliferation. (A)** Daoy and UW228 cells transfected with a siRNA targeting *WEE1* (siWEE1) displayed a decrease in cell proliferation when compared to a non-silencing siRNA (siRNA N.C.). Forty-eight hours after transfection cells were seeded into E-plates and real-time cell proliferation monitored using the xCELLigence system. **(B)** Transfection with siWEE1 significantly decreased the relative colony number compared to siRNA N.C. Representative pictures for each treatment group are above the quantifying bar graphs. **(C)** RNAi-mediated inhibition of *WEE1* decreased the levels of WEE1 protein in both the Daoy and UW228 cell lines 72 hours after transfection.

Daoy and UW228 cell lines were chosen to examine the functional consequence of inhibiting *WEE1*. Using a siRNA against *WEE1* we measured cell proliferation using the xCELLigence real-time cell analysis (RTCA) system. Cell growth was monitored following transfection of either a non-silencing siRNA or one targeting *WEE1*. A decrease in cell growth was seen in both the Daoy and UW228 cell lines (Figure 3A). The more pronounced decrease in growth seen in Daoy cells compared to UW228 cells could be explained by the higher expression of WEE1 protein in Daoy cells compared to the UW228 cell line (Figure 2E). We then chose to determine the ability of medulloblastoma cells to undergo an unlimited number of divisions following inhibition of *WEE1* by performing a colony forming assay. The siRNA targeting *WEE1* showed a significant decrease in the relative colony number when compared to the non-silencing siRNA in both Daoy and UW228 cell lines (Figure 3B). Western blots confirmed the specificity and ability of the siRNA targeting *WEE1* to decrease the WEE1 protein levels in Daoy and UW228 cells (Figure 3C).

### Small molecule inhibitor of WEE1, MK-1775, potently suppresses medulloblastoma growth in vitro and in vivo

**Figure 4:**
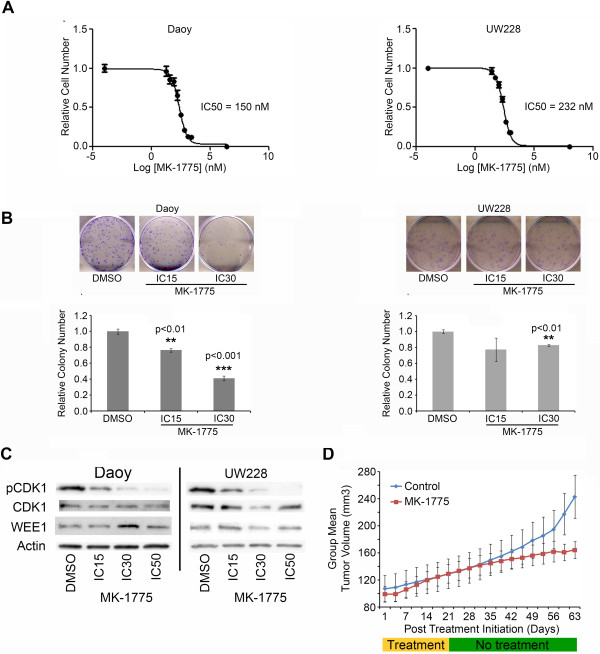
**Inhibition of WEE1 by the small molecule inhibitor MK-1775. (A)** A decrease in relative cell number was seen by MTS assay through a wide range of MK-1775 concentrations in both Daoy and UW228 cells. From this data the IC50 values were calculated. **(B)** Treatment with an IC30 of MK-1775 for 48 hours significantly decreased the relative colony numbers in Daoy and UW228 cells. Shown are representative images from each treatment group with the quantifying bar graph for each cell line below the images. **(C)** Exposure to increasing concentrations of MK-1775 in Daoy and UW228 cells showed a dose dependent decrease in WEE1 activity as seen by decreased phospho-CDK1 (Tyr15). There was no significant change in WEE1 levels. **(D)** Treatment with MK-1775 is sufficient to decrease subcutaneous tumor growth of Daoy cells in mice. Mice were treated on three consecutive days a week for the first three weeks after tumor establishment.

Recently several inhibitors of WEE1 have been described [28-30]. We chose to examine MK-1775, a pyrazolo-pyrimidine derivative, which has recently entered Phase I/II trials in adult cancers. Daoy and UW228 cells were exposed to various concentrations of MK-1775. The IC50 values from the MTS assay were 150 nM and 232 nM for Daoy and UW228 cell lines respectively (Figure 4A). These nanomolar concentrations have been shown previously to be achievable *in vivo* and therefore make MK-1775 a clinically viable drug [31].

To evaluate the impact of MK-1775 on the long-term proliferative capacity in medulloblastoma cells, colony forming assays were performed in both Daoy and UW228 cell lines. In both cell lines 48 hour exposure to an IC30 of MK-1775 was sufficient to significantly decrease the colony number counted after 1 week (p < 0.01, Figure 4B). Furthermore, the Daoy cell line showed a significant decrease in relative colony number after exposure to an IC15 of MK-1775 (p < 0.01). UW228 cells did not show a further decrease in colony formation from the IC30 to IC15, which likely reflects the heterogeneity of tumor cells. Interestingly UW228 are p53 sufficient while Daoy cells are not. Clearly the role of p53 status on MK-1775 activity in medulloblastoma needs to be further examined. To assess whether MK-1775 was inhibiting WEE1 kinase activity, we measured the phosphorylation status of its target CDK1 by immunoblotting. A decrease in phospho-CDK1 (Tyr15) was seen in a dose-dependent manner with MK-1775 treatment as shown in Figure 4C. There was no significant change in WEE1 with MK-1775 treatment as expected.

We next examined the efficacy of MK-1775 in treating medulloblastoma tumors *in vivo*. Athymic nude mice were injected with Daoy cells subcutaneously and palpable tumors were allowed to develop. Mice were treated with an oral gavage of either control solution or 30 mg/kg of MK-1775 three times a week for three weeks (n = 10 each group). MK-1775 was able to decrease the measurable size of the tumors compared to controls as seen in Figure 4D (p < 0.005). A decrease in tumor volume was observed on day 63 at the termination of the study (Additional file 4: Figure S2A, p < 0.01). Recent evidence suggests that inhibition of *WEE1* can lead to DNA damage. To determine if the decrease in tumor volume may have resulted from increased DNA damage snap frozen tumors were probed for γH2AX, a marker of DNA double strand breaks. An increase in γH2AX protein was seen in the MK-1775 treated tumors when compared to the control tumors at day 63 (Additional file 4: Figure S2B). There was no change in the phosphorylation of CDK1, WEE1 or in the total levels of CDK1 or WEE1 in the tumor samples (data not shown) as expected. The tumors had only been treated with MK-1775 during the initial part of the study and we would not expect a transient marker like the phosphorylation of CDK1 to persist. These studies establish the proof of principle that MK-1775 is a viable candidate for medulloblastoma therapy. These data are further supported by recent studies demonstrating the single agent activity of MK-1775 in many different tumor cell lines [32].

### MK-1775 acts in synergy with cisplatin

**Figure 5:**
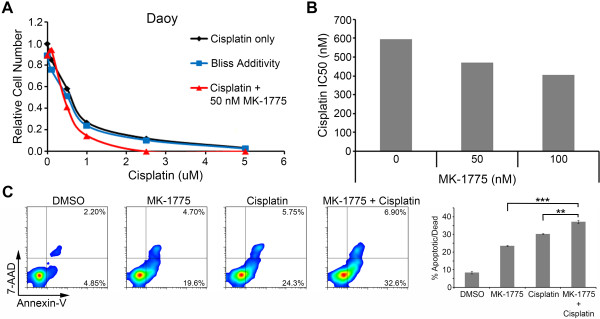
**MK-1775 acts synergistically with cisplatin. (A)** Line graph depicting the effect of different cisplatin doses with or without treatment of MK-1775 on Daoy relative cell number by MTS assay. The black line represents cells treated only with cisplatin. The blue line depicts the calculated effect of cisplatin and 50 nM MK-1775 using the Bliss Additivity model. The red line represents the observed effect of cisplatin and 50 nM MK-1775 combination treatments. **(B)** The bar graph depicts the calculated IC50 value by MTS assay of cisplatin in Daoy cells exposed only to cisplatin (0 nM MK-1775) or in combination with 50 nM MK-1775 or 100 nM MK-1775. **(C)** Representative flow cytometry plots depict the effect on apoptosis resulting from 48 hours of drug treatment in Daoy cells following staining with Guava Nexin reagent. The lower right and upper right quadrants show cells that are Annexin V positive and thus considered apoptotic or dead. The bar graph on the right quantifies the average percent of cells apoptotic or dead in these two quadrants for the replicate samples. The combination of an IC25 of cisplatin with an IC30 of MK-1775 increased the percentage of apoptotic cells. **p < 0.01; ***p < 0.001.

Cisplatin forms the backbone of most medulloblastoma chemotherapy protocols. Among the adverse effects of cisplatin are cytotoxicity, nephrotoxicity and severe bone marrow suppression. Thus drugs that can synergize with cisplatin and facilitate a decrease in this chemotherapy are of high value. We examined the ability of MK-1775 to synergize with cisplatin. Daoy cells were exposed to varying concentrations of MK-1775 and cisplatin for 72 hours and the effect on cellular proliferation was evaluated by MTS assay. The results were analyzed using the Bliss Additivity model and show a synergistic relationship between cisplatin and MK-1775 at a concentration as low as 50 nM of MK-1775 (Figure 5A). The effect of MK-1775 on the IC50 value for cisplatin was also investigated using Daoy cells. Daoy cells have an IC50 value of 593 nM for cisplatin alone by MTS assay. Figure 5B shows a decrease in the IC50 value of cisplatin down to 470 nM and 406 nM when Daoy cells were treated with cisplatin and either 50 nM or 100 nM of MK-1775 respectively.

### MK-1775 induces caspase-mediated apoptosis in medulloblastoma

To determine if the suppression of cellular growth was a result of cell death, we evaluated the impact of MK-1775 on apoptosis in medulloblastoma cells. The Daoy cell line was exposed to an IC30 of MK-1775, an IC25 of cisplatin, or both for 48 hours. The cells were subsequently stained with Guava Nexin reagent for Annexin V expression and analyzed using a flow cytometer. Treatment with MK-1775 alone for 48 hours caused an increase in apoptotic cells (Figure 5C). Moreover, a significant increase in the percentage of Annexin V positive cells was seen when cells were treated with both MK-1775 and cisplatin compared to treatment with either drug alone (p < 0.01). The increase in apoptotic cells can also be seen by increased caspase 3 staining in the immunofluorescence images in Additional file 5: Figure S3.

### MK-1775 treatment produces DNA damage and inhibits repair of cisplatin-induced DNA damage

**Figure 6:**
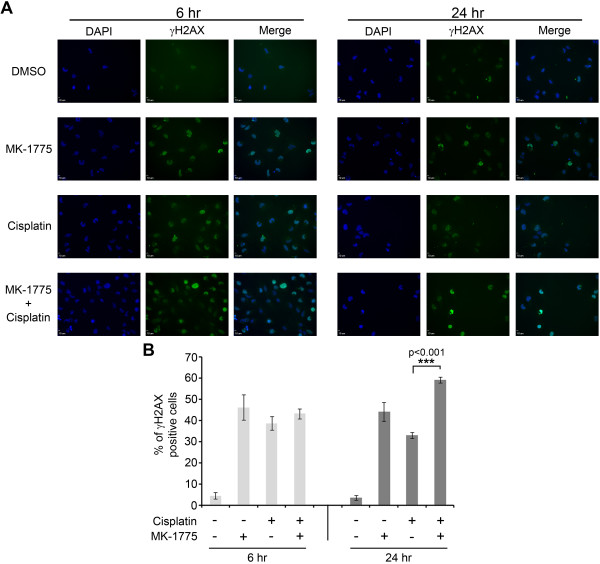
**Cisplatin-induced DNA damage is increased with MK-1775 treatment. (A)** Immunofluorescence images of Daoy cells treated with an IC30 of MK-1775 or an IC25 of cisplatin or both drugs for the time periods as indicated. Cells were stained with DAPI (blue) for total DNA content and with an antibody against γH2AX (green) to visualize DNA damage. **(B)** Quantifying bar graph showing the normalized percentage of γH2AX positive Daoy cells co-stained with DAPI nuclear cells.

The DNA damage pathway plays an important part for the survival of tumor cells, especially after treatment with DNA damaging agents. To examine the impact of MK-1775 on medulloblastoma cells, we analyzed for DNA damage by staining with an antibody against γH2AX and co-staining of the nuclei with DAPI (Figure 6A). γH2AX is recruited to sites of damaged DNA and is removed once DNA is repaired. After 6 hours of treatment with an IC30 of MK-1775 and/or an IC25 of cisplatin both significantly induced DNA damage as measured by γH2AX foci in Daoy cells (Figure 6A). However there was no significant difference between the percentages of γH2AX positive cells in cisplatin alone or MK-1775 treated cells for 6 hours (Figure 6B). We then measured existing DNA damage after treatment with cisplatin, MK-1775 or both for 24 hours. While there was a decrease in γH2AX foci in cisplatin alone treated cells compared to 6 hour treatment, there was an increase in γH2AX foci in cells treated with both MK-1775 and cisplatin. These data indicate that adding MK-1775 inhibits DNA damage repair seen after 24 hours of treatment with cisplatin alone.

## Discussion

Medulloblastoma is a clinically and genomically heterogeneous disease. This heterogeneity creates a major challenge in developing novel therapy and improving outcomes in medulloblastoma using traditional approaches. Thus, novel approaches that more rapidly identify and validate mediators of cell survival have the potential to dramatically alter our understanding of cellular biology and develop novel therapeutic strategies. We hypothesized that escape pathways on which medulloblastoma cells are dependent for survival would represent ideal targets for therapy. To identify these pathways we integrated descriptive and functional genomics to objectively elucidate new targets for rationale therapy development. We identified a medulloblastoma specific kinase expression profile that can provide insight into future kinase inhibition therapeutic strategies. Next using RNAi functional genomics targeting the kinome we identified key molecules that mediate medulloblastoma cell proliferation. Integration of these data suggests that the mitotic cell cycle checkpoint is a critical determinant of medulloblastoma tumorigenesis. In particular we identified *WEE1* as a potential therapeutic target. A combination of *in vitro* and *in vivo* studies clearly indicates that *WEE1* mediates medulloblastoma tumorigencity and represents a novel therapeutic target. These data demonstrate the power of integrating descriptive and functional genomics to identify novel therapeutic targets. Using a similar kinome-wide RNAi screen Guerreiro et al., previously identified PI3K p110γ as a molecule that demonstrated synthetic lethality with cisplatin in medulloblastoma [33]. Together with our data these two studies demonstrate the utility of functional genomics to identify novel therapeutic targets in medulloblastoma.

Cell cycle checkpoint genes have previously been implicated in medulloblastoma [34-36]. However to our knowledge this is the first report in medulloblastoma to document the functional importance of mitotic kinases and in particular *WEE1*. WEE1 controls the G2-M transition by catalyzing the inhibitory phosphorylation of CDK1 thus preventing the Cyclin B-CDK1 complex from driving cells into mitosis [37]. The G2-M cell cycle checkpoint is critical for eukaryotic organisms ensuring cells do not initiate mitosis before DNA damage is repaired [38]. *WEE1* is a key regulator of this process [37,28]. Thus abrogation of the G2-M checkpoint leading to premature mitotic entry and subsequent cell death by mitotic catastrophe has emerged as a promising new therapeutic strategy [39,38].

*WEE1* over expression is associated with several types of cancer [38]. More recently an *in silico* analysis of a large data set of glioblastoma identified *WEE1* as over expressed and a key regulator of mitotic catastrophe [27]. Similarly a functional genomic approach in acute myeloid leukemia also identified *WEE1* as a key regulator of chemotherapy sensitivity [40]. This study shows that *WEE1* is over expressed and functionally important in medulloblastoma. Our data show that the small molecule inhibitor of WEE1, MK-1775, is a potent inhibitor of medulloblastoma cell growth and synergizes with cisplatin to decrease cell proliferation *in vitro*. Cisplatin in combination with MK-1775 treatment induces an increase in the percentage of cells in the S and G2-M phases of the cell cycle (Additional file 6: Figure S4A) and subsequently leads to more DNA damage as measured by γH2AX foci than DNA damage induced by cisplatin alone. Similarly an increase in mitosis was demonstrated in the combination treatment by increased phospho-H3 staining as seen in Additional file 6: Figure S4B and C. Interestingly, MK-1775 as a single agent potently inhibits medulloblastoma tumor growth *in vivo*. These data could be explained by the fact that MK-1775 induces DNA damage and genomic instability. MK-1775 has recently been shown to inhibit growth of sarcoma cells and glioblastoma cells among many other tumor types [31,41,42].

Inhibition of WEE1 has largely been investigated in the context of abnormal p53 function, given that cells with impaired p53 function are highly dependent on the G2-M checkpoint to maintain genomic integrity [43]. For example studies have demonstrated that cells with dysfunctional p53 can be sensitized to DNA damage by impairing the G2-M checkpoint through inhibition of WEE1 [30]. Our data showing synergy of WEE1 inhibition in combination with cisplatin were generated in a medulloblastoma cell line that has nonfunctional p53. A more thorough investigation with additional functional p53, nonfunctional p53 and isogenic cell lines are needed to determine the role of p53 on the effectiveness of WEE1 inhibition to sensitize cells to DNA damaging agents.

We have demonstrated that WEE1 inhibition sensitizes medulloblastoma cells to cisplatin *in vitro*. However it is unlikely that all patients with medulloblastoma will respond in a similar manner. Thus, it will be important to develop suitable biomarkers to predict which patients may benefit the most from such a therapeutic strategy. Detailed animal modeling of medulloblastoma with assessment of potential biomarkers as well as pharmacokinetics will provide further data in evaluating the effectiveness of WEE1 inhibition in conjunction with cisplatin in eradicating medulloblastoma cells *in vivo* and prolonging patient survival.

Importantly, MK-1775 is a well-tolerated drug with low dose limiting toxicities [44]. Clinical trials are now underway for MK-1775 including combination with carboplatin for ovarian cancer (NCT01164995) and combination with gemcitabine, cisplatin or carboplatin in advanced solid tumors (NCT00648648). Because MK-1775 crosses the blood brain barrier it is a promising agent for brain tumor therapy. In fact a Phase I trial in glioblastoma multiforme in adults is currently underway (NCT01849146) as well as a newly initiated trial for diffuse intrinsic pontine glioma in children (NCT01922076). For medulloblastoma therapy we envision a strategy of administering MK-1775 thrice weekly for 3 weeks with each cycle of cisplatin. Thus once the maximum tolerated dose is obtained in a Phase I trial, we would propose a Phase II/III trial of cisplatin based therapy (the current standard of care) compared to cisplatin plus MK-1775.

In summary our data supports a role for WEE1 in regulating the G2-M checkpoint in medulloblastoma and validated WEE1 as a therapeutic target. We demonstrate that a clinically relevant small molecule inhibitor, MK-1775, is potent in the inhibition of medulloblastoma tumor growth *in vivo*. In light of our data and that of others, in combination with the good positive safety profile, we suggest that MK-1775 is an exciting new agent in the treatment of pediatric brain tumors, particularly medulloblastoma.

## Competing interest

The authors declare that they have no competing interests.

## Authors’ contribution

PSH: Performed siRNA screening, designed experiments and wrote the manuscript. SV: Performed immunofluorescence assays and assisted in reviewing the manuscript. IA: Performed cell proliferation assays. DKB and BC: Performed computational analysis. IB: Performed immunoblotting. AMD and MDT: Obtained and analyzed the second large cohort of medulloblastoma samples. PR: Synthesized MK-1775. NKF and RV: obtained medulloblastoma patient samples, co-designed and conceived experiments and wrote paper. All authors read and approved the final manuscript.

## Supplementary Material

Additional file 1: Table S1. Dysregulated cell cycle-related kinases in medulloblastoma. Click here for file

Additional file 2: Table S2. Genes that attenuate medulloblastoma cell growth. Click here for file

Additional file 3: Figure S1. Inhibition of cell cycle-related kinases decreased Daoy cell proliferation. Shown are five select kinases demonstrating a decrease in medulloblastoma proliferation by MTS assay. The bar graphs show the effect of the three separate siRNAs from the kinase screen on relative cell number after being normalized to the non-silencing control siRNA (siRNA N.C.). ***p < 0.001. Click here for file

Additional file 4: Figure S2. MK-1775 treatment decreases tumor volume and increases DNA damage in Daoy xenografts. (A) The graph depicts the average subcutaneous tumor volume in mice treated with the vehicle control DMSO or MK-1775 at the termination of the study. (B) Western blot analysis of tumors isolated at the termination of the study demonstrates an increase in γH2AX protein for mice treated with MK-1775 when compared to control. Click here for file

Additional file 5: Figure S3. MK-1775 induces caspase 3 activation in medulloblastoma cells. Immunofluorescence images of Daoy cells treated with an IC30 of MK-1775 or an IC25 of cisplatin or both drugs are shown. Total DNA content is visualized by blue DAPI staining and caspase 3 is shown by green staining. Click here for file

Additional file 6: Figure S4. Combination treatment with MK-1775 and cisplatin increases mitosis in Daoy cells. (A) Cell cycle analysis of Daoy cells treated with an IC30 of MK-1775, an IC25 of cisplatin or both for 24 hours. An increase in the percentage of cells in S and G2-M phases was observed with combination treatment. (B) Representative immunofluorescence images of Daoy cells treated with an IC30 of MK-1775, an IC25 of cisplatin, or both for 24 hours. Blue DAPI staining demonstrates nucleated cells and green staining depicts phospho-H3. (C) Quantitation of phospho-H3 positive cells in each treatment group normalized to DAPI. Click here for file
