# Supplementary material for: Integrated genomic analysis identifies the mitotic checkpoint kinase WEE1 as a novel therapeutic target in medulloblastoma
Source: Mol Cancer. 2014 Mar 24;13:72. doi: 10.1186/1476-4598-13-72 (PMC3987923; doi:10.1186/1476-4598-13-72)
Supplement: Additional file 1: Table S1. — Dysregulated cell cycle-related kinases in medulloblastoma. [file 1476-4598-13-72-S1.doc]

|  | **Genes Down in MB** | | **Genes Up in MB** | |
| --- | --- | --- | --- | --- |
|  | PRKACA |  | | BUB1B |
|  | CAMK2D |  | | PLK4 |
|  | MAPK4 |  | | MELK |
|  | RPS6KA1 |  | | TTK |
|  | PRKCE |  | | NEK2 |
|  | FGFR1 |  | | CDK1 |
|  | STK10 |  | | PBK |
|  | CIT |  | | AURKA |
|  | DGKZ |  | | WEE1 |
|  | AKT2 |  | | CHEK1 |
|  | FGFR2 |  | | CCNB1 |
|  | MAPK1 |  | | CDK2 |
|  | MAP2K1 |  | | MASTL |
|  | RPS6KA2 |  | | CDK4 |
|  | CAMK2B |  | | E2F3 |
|  | DLG1 |  | | BUB1 |
|  | EGFR |  | | TGFBR1 |
|  | UHMK1 |  | | CDK6 |
|  | CCND3 |  | | AURKB |
|  | CAMK1 |  | | SKP2 |
|  | MAP3K11 |  | | CKS1B |
|  |  |  | | RB1 |
|  |  |  | | PKMYT1 |
|  |  |  | | CSNK2A2 |
|  |  |  | | CHEK2 |
|  |  |  | | VRK1 |
|  |  |  | | CSNK1E |
|  |  |  | | PLK2 |
|  |  |  | | ATM |

Additional file 1: Table S1
