# Supplementary material for: Integrated genomic analysis identifies the mitotic checkpoint kinase WEE1 as a novel therapeutic target in medulloblastoma
Source: Mol Cancer. 2014 Mar 24;13:72. doi: 10.1186/1476-4598-13-72 (PMC3987923; doi:10.1186/1476-4598-13-72)
Supplement: Additional file 2: Table S2. — Genes that attenuate medulloblastoma cell growth. [file 1476-4598-13-72-S2.doc]

| **Gene** | **Avg Z score** |
| --- | --- |
| AURKA | -41.4952 |
| NEK2 | -37.7765 |
| WEE1 | -37.6346 |
| TTK | -24.1675 |
| MAPK14 | -23.1761 |
| CHKA | -19.5612 |
| PRKD3 | -17.7759 |
| PIK3R4 | -17.741 |
| CDK2 | -16.4429 |
| ADRBK2 | -16.0782 |
| PLK1 | -14.662 |
| AK3L1 | -14.5823 |
| RIOK2 | -13.7697 |
| MST1R | -7.19223 |
| FES | -6.10805 |
| GAK | -6.00036 |
| CDK10 | -5.80499 |
| MAP3K4 | -5.54081 |
| CHEK1 | -5.46191 |
| SIK1 | -5.4525 |
| ASB10 | -5.33952 |
| CKMT1B | -5.2035 |
| IKBKB | -4.9507 |
| STK38 | -4.53395 |
| MAP4K5 | -4.5262 |
| IKBKG | -4.45202 |
| PRKCD | -4.39471 |
| MATK | -4.3849 |
| PIP5K1B | -4.35175 |
| BCKDK | -4.34231 |
| ERBB2 | -4.2015 |
| DDR1 | -4.10015 |
| TEX14 | -4.02631 |
| VRK3 | -3.82566 |
| AK2 | -3.80608 |
| DCK | -3.79663 |
| AKAP14 | -3.71124 |
| NTRK2 | -3.64507 |
| CAMK2D | -3.62002 |
| ALPK2 | -3.56935 |
| CKMT2 | -3.55998 |
| FRAP1 | -3.54553 |
| PIP5K1A | -3.48341 |
| ANKK1 | -3.4535 |
| AK3L2 | -3.41239 |
| LMTK2 | -3.34484 |
| DCLK3 | -3.3158 |
| MPP4 | -3.2391 |
| NLK | -3.02449 |
| C1orf57 | -3.02006 |
| GRK5 | -2.95491 |
| NEK10 | -2.93654 |
| GSK3B | -2.89074 |
| MLKL | -2.85359 |
| DCAKD | -2.81949 |
| EIF2AK2 | -2.80522 |
| C19orf35 | -2.80104 |
| RPS6KB2 | -2.7807 |
| ABL2 | -2.74707 |
| RIOK1 | -2.74607 |
| CAMKK2 | -2.66354 |
| CDKL1 | -2.65785 |
| CERKL | -2.65153 |
| PFTK1 | -2.63189 |
| PHKG2 | -2.58817 |
| TAOK3 | -2.57323 |
| PFKP | -2.5602 |
| DGKK | -2.55888 |
| PIK3CD | -2.5587 |
| PDPK1 | -2.54427 |
| SIK2 | -2.53445 |
| MST4 | -2.50631 |
| TRRAP | -2.50129 |
| NEK6 | -2.49167 |
| EIF2AK4 | -2.45929 |
| HK1 | -2.44582 |
| LRRK2 | -2.44099 |
| CDC42SE2 | -2.39208 |
| PFKL | -2.37932 |
| MARK3 | -2.34234 |
| STK25 | -2.34047 |
| WNK1 | -2.31287 |
| ALK | -2.30373 |
| PFKM | -2.29221 |
| ALPK1 | -2.24403 |
| PRKD1 | -2.21741 |
| PIP5K1C | -2.16896 |
| MAP3K3 | -2.15013 |
| PGK1 | -2.14698 |
| AKT3 | -2.12703 |
| MAP3K14 | -2.10443 |
| PI4KB | -2.09713 |
| CLK2 | -2.09117 |
| STAP1 | -2.07357 |
| PAK1 | -2.0005 |

Additional file 2: Table S2
