# Supplementary figures and images for: Integrated genomic analysis identifies the mitotic checkpoint kinase WEE1 as a novel therapeutic target in medulloblastoma
Source: Mol Cancer. 2014 Mar 24;13:72. doi: 10.1186/1476-4598-13-72 (PMC3987923; doi:10.1186/1476-4598-13-72)

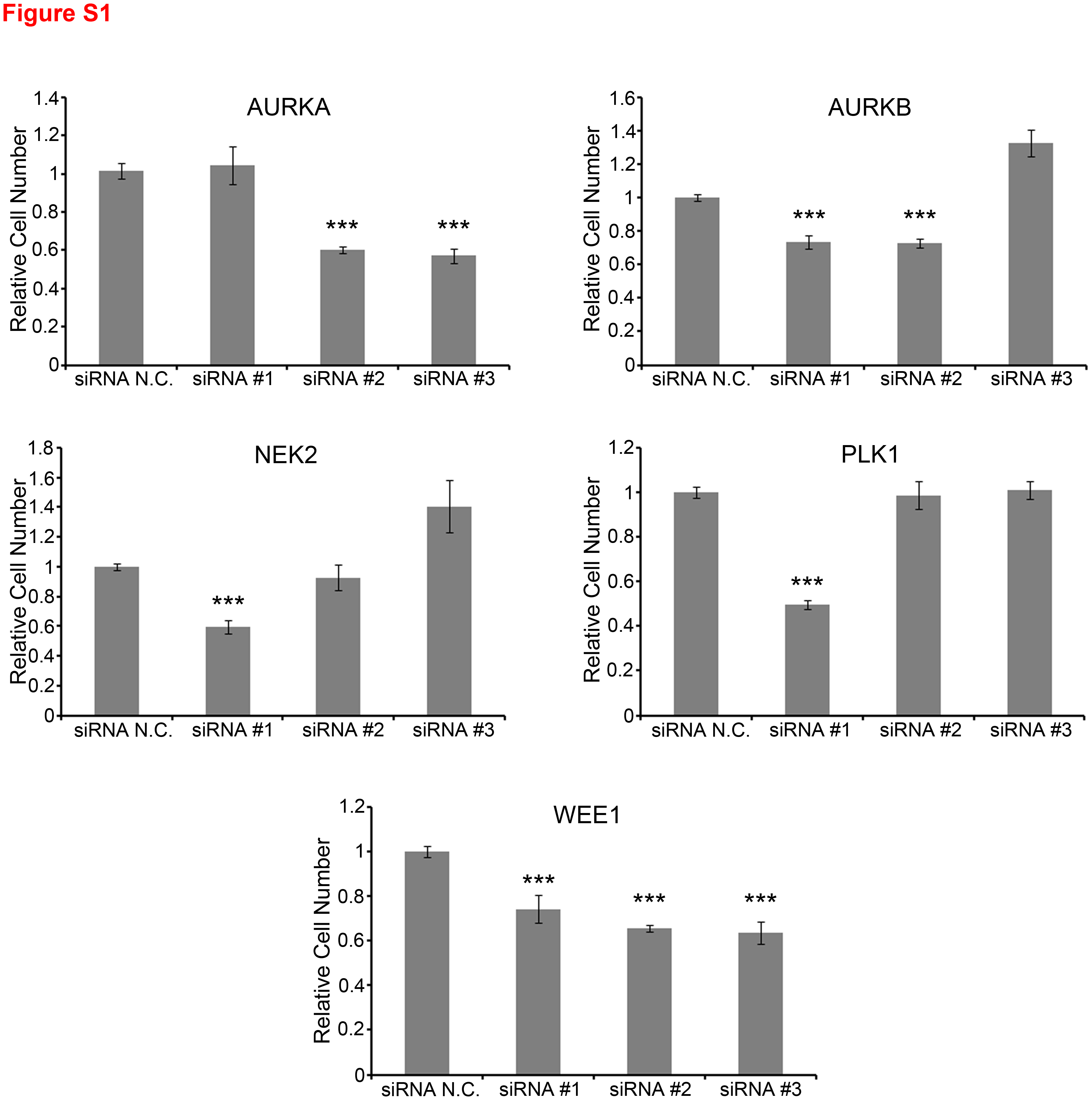

Supplement: Additional file 3: Figure S1. — Inhibition of cell cycle-related kinases decreased Daoy cell proliferation. Shown are five select kinases demonstrating a decrease in medulloblastoma proliferation by MTS assay. The bar graphs show the effect of the three separate siRNAs from the kinase screen on relative cell number after being normalized to the non-silencing control siRNA (siRNA N.C.). ***p < 0.001. [file 1476-4598-13-72-S3.png]

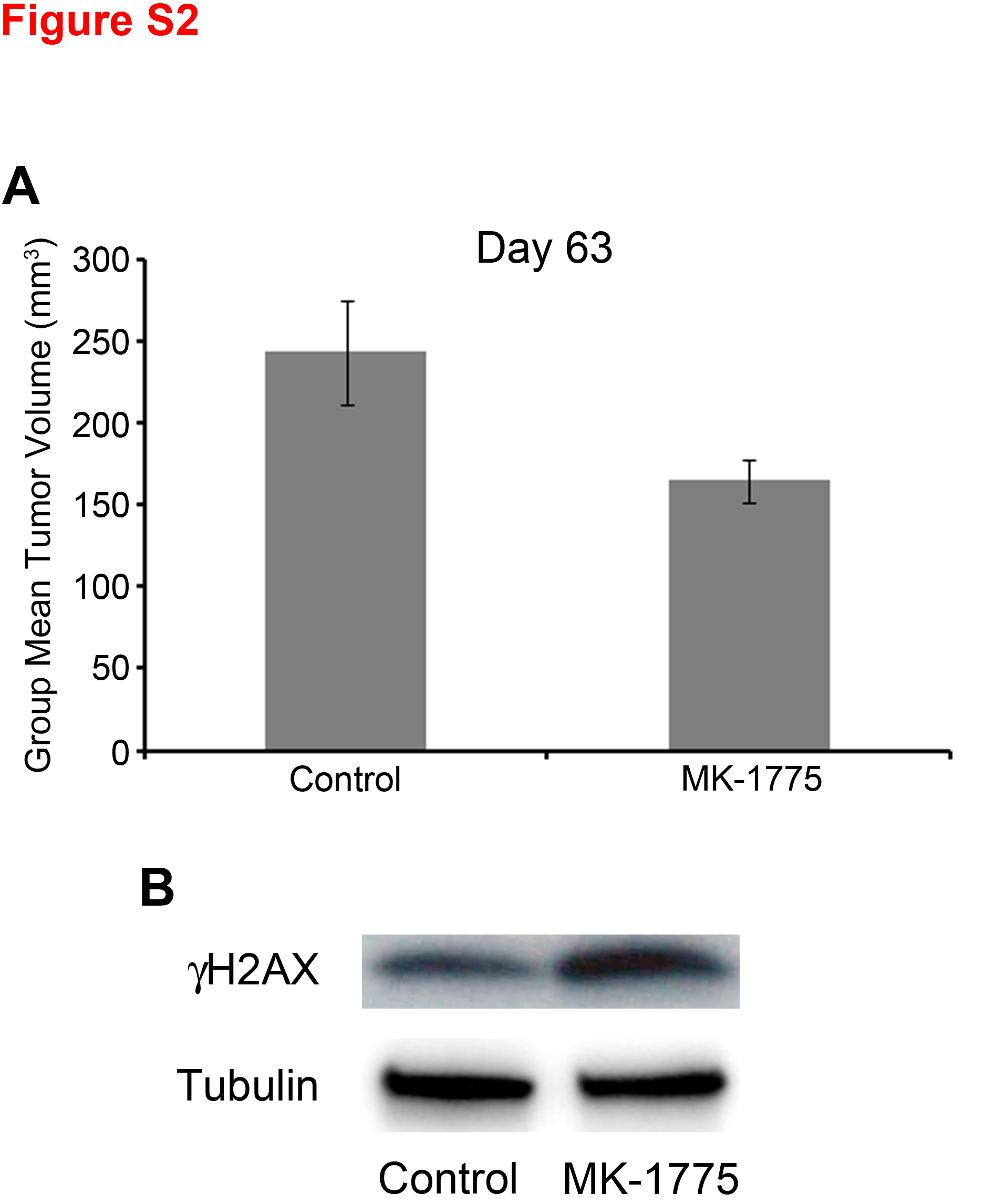

Supplement: Additional file 4: Figure S2. — MK-1775 treatment decreases tumor volume and increases DNA damage in Daoy xenografts. (A) The graph depicts the average subcutaneous tumor volume in mice treated with the vehicle control DMSO or MK-1775 at the termination of the study. (B) Western blot analysis of tumors isolated at the termination of the study demonstrates an increase in γH2AX protein for mice treated with MK-1775 when compared to control. [file 1476-4598-13-72-S4.png]

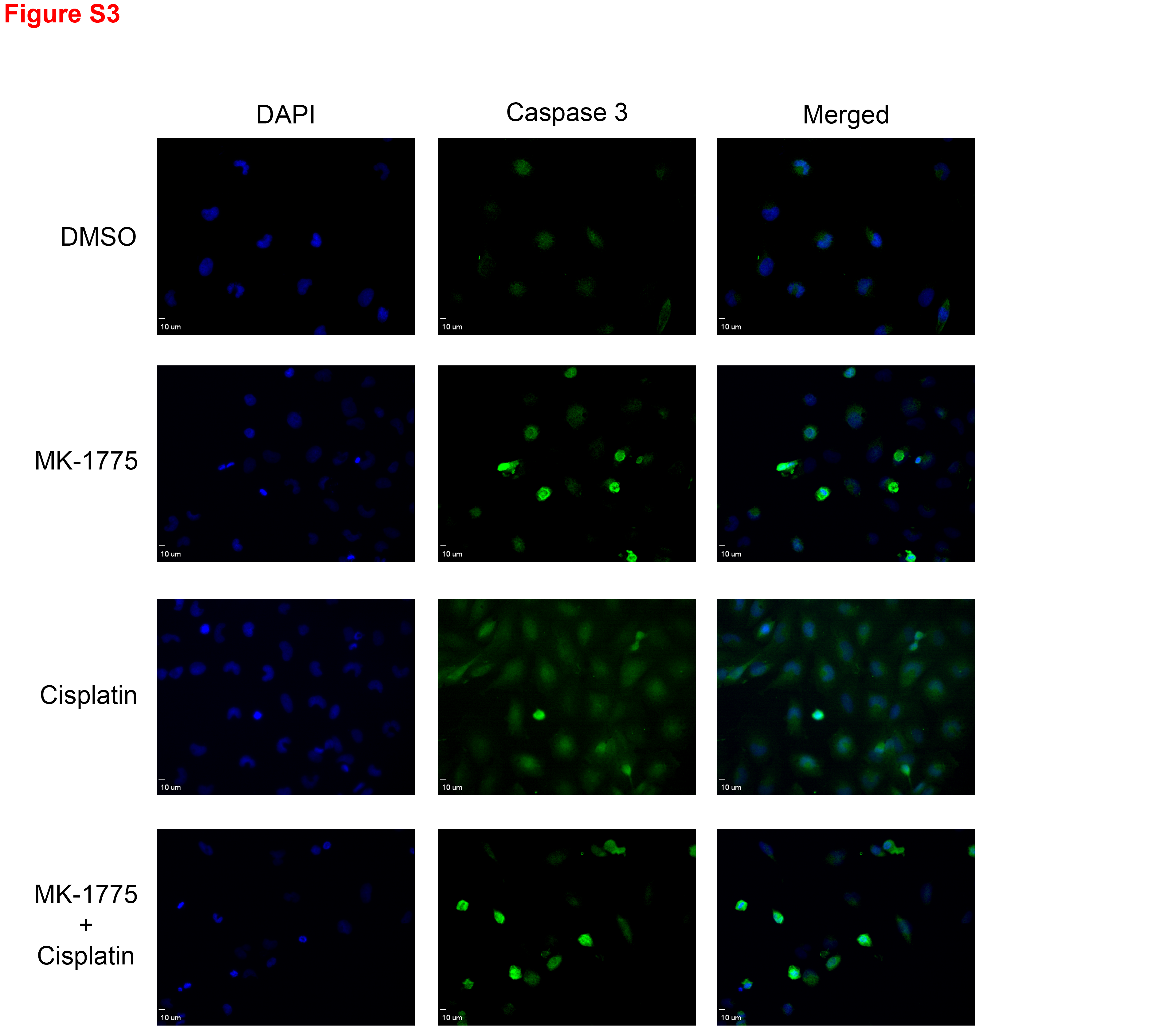

Supplement: Additional file 5: Figure S3. — MK-1775 induces caspase 3 activation in medulloblastoma cells. Immunofluorescence images of Daoy cells treated with an IC30 of MK-1775 or an IC25 of cisplatin or both drugs are shown. Total DNA content is visualized by blue DAPI staining and caspase 3 is shown by green staining. [file 1476-4598-13-72-S5.png]

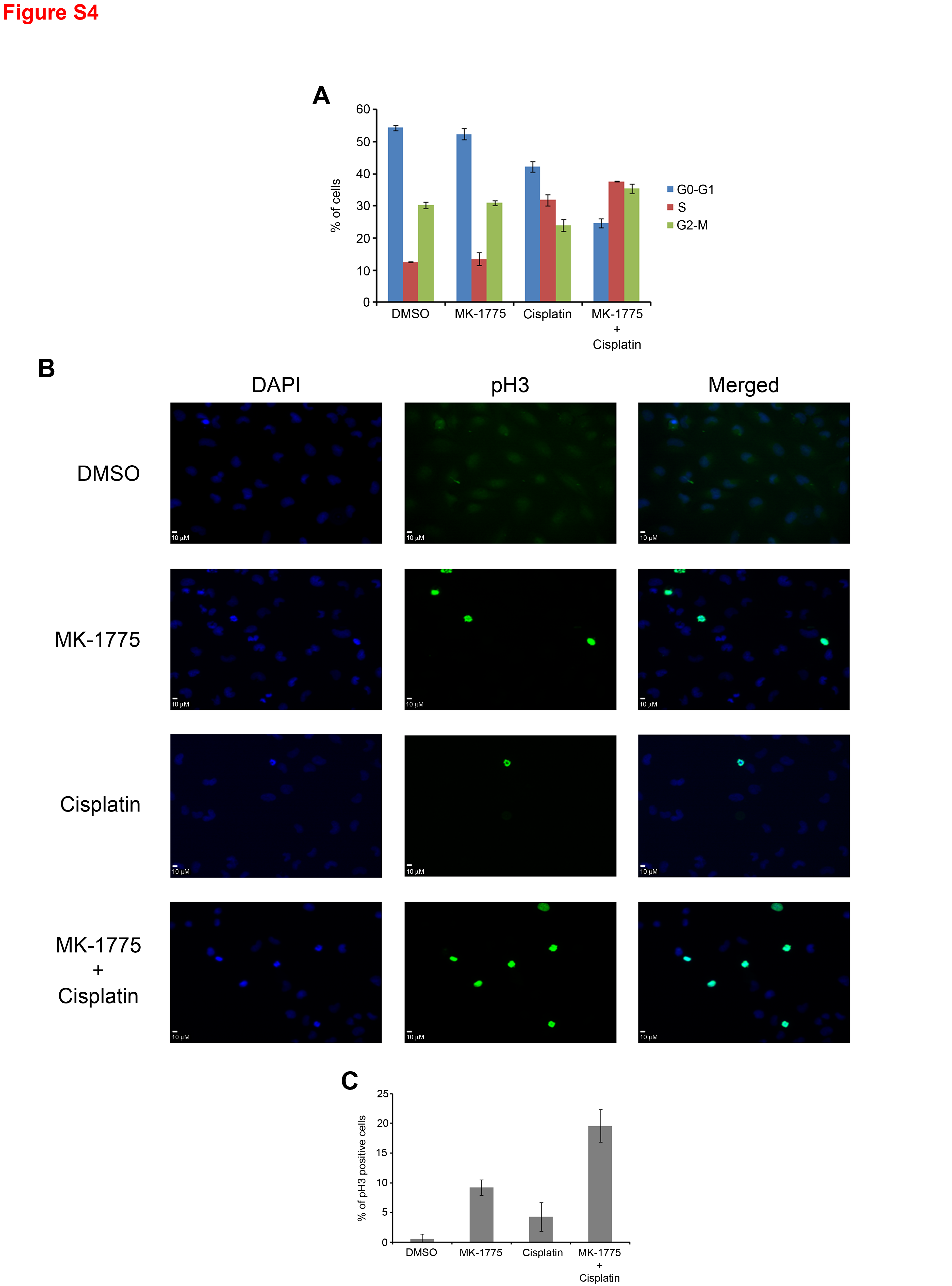

Supplement: Additional file 6: Figure S4. — Combination treatment with MK-1775 and cisplatin increases mitosis in Daoy cells. (A) Cell cycle analysis of Daoy cells treated with an IC30 of MK-1775, an IC25 of cisplatin or both for 24 hours. An increase in the percentage of cells in S and G2-M phases was observed with combination treatment. (B) Representative immunofluorescence images of Daoy cells treated with an IC30 of MK-1775, an IC25 of cisplatin, or both for 24 hours. Blue DAPI staining demonstrates nucleated cells and green staining depicts phospho-H3. (C) Quantitation of phospho-H3 positive cells in each treatment group normalized to DAPI. [file 1476-4598-13-72-S6.png]
